# Nitrosyl-hemoglobin formation in rodent and human venous erythrocytes reflects NO formation from the vasculature in vivo

**DOI:** 10.1371/journal.pone.0200352

**Published:** 2018-07-11

**Authors:** Flavia Dei Zotti, Irina I. Lobysheva, Jean-Luc Balligand

**Affiliations:** Institut de Recherche Experimentale et Clinique (IREC), Pole of Pharmacology and Therapeutics (FATH), Cliniques Universitaires Saint-Luc and Université Catholique de Louvain, Brussels, Belgium; Institut d'Investigacions Biomediques de Barcelona, SPAIN

## Abstract

Reduced bioavailability of nitric oxide (NO) is a major feature of endothelial dysfunction characteristic of cardiovascular and metabolic diseases but the short half-life of NO precludes its easy quantification in circulating blood for early diagnosis. In erythrocytes, NO can react with hemoglobin to form an iron-nitrosyl complex (5-coordinate-α-HbNO) directly quantifiable by Electron Paramagnetic Resonance spectroscopy (EPR) in mouse, rat and human venous blood *ex vivo*. However, the sources of the nitrosylating species *in vivo* and optimal conditions of HbNO preservation for diagnostic use in human erythrocytes are unknown. Using EPR spectroscopy, we found that HbNO stability was significantly higher under hypoxia (equivalent to venous pO_2_; 12.0±0.2% degradation of HbNO at 30 minutes) than at room air (47.7±0.2% degradation) in intact erythrocytes; at 20°C (15.2±0.3% degradation after 30 min versus 29.6±0.1% at 37°C) and under acidic pH (31.7±0.8% versus 62.2±0.4% degradation after 30 min at physiological pH) at 50% of haematocrit. We next examined the relative contribution of NO synthase (NOS) from the vasculature or in erythrocytes themselves as a source of nitrosylating NO. We detected a NOS activity (and eNOS expression) in human red blood cells (RBC), and in RBCs from eNOS^(+/+)^ (but not eNOS^(-/-)^) mice, as measured by HbNO formation and nitrite/nitrate accumulation. NO formation was increased after inhibition of arginase but abrogated upon NOS inhibition in human RBC and in RBCs from eNOS^(+/+)^ (but not eNOS^(-/-)^) mice. However, the HbNO signal from freshly drawn venous RBCs was minimally sensitive to the inhibitors *ex vivo*, while it was enhanced upon caveolin-1 deletion *in vivo*, suggesting a minor contribution of erythrocyte NOS to HbNO complex formation compared with vascular endothelial NOS or other paracrine NO sources. We conclude that HbNO formation in rodent and human venous erythrocytes is mainly influenced by vascular NO sources despite the erythrocyte NOS activity, so that its measurement by EPR could serve as a surrogate for NO-dependent endothelial function.

## Introduction

Nitric oxide (NO) availability in the vasculature determines the efficiency of key functions of endothelial cells, such as vasodilation, angiogenesis and thrombosis inhibition [1–3]. Endothelial dysfunction with impaired levels of NO availability is the initiating step towards the development of cardiovascular and metabolic diseases in association with hypertension, atherosclerosis, diabetes, hypercholesterolemia and aging [4, 5]. Vascular NO is produced in endothelial cells by one of the NO synthase isoforms (eNOS) from its substrate L-arginine in an enzymatic reaction that requires molecular oxygen, NADPH and redox cofactors [6]. The expression of an eNOS-like enzyme was also demonstrated in erythrocytes, the activity of which could be unveiled upon inhibition of arginase-1, an enzyme competing with eNOS for their common substrate L-arginine [7–9]. However, the effects of the erythrocyte NO on target tissues upon potential NO export are still under debate [8–13]. Regardless, any measurement of the bioavailable NO in the human circulation *in vivo* would be highly desirable to monitor the progression of cardiovascular diseases, but remains a challenge due to low NO stability and various processes influencing NO diffusion to potential targets. Electron Paramagnetic Resonance (EPR) spectroscopy, a method for quantitative detection of different paramagnetic compounds in biological samples, has been proposed over the last decades as a means to achieve that [14, 15]. One of the paramagnetic NO adducts is heme-nitrosylated hemoglobin formed in erythrocytes when NO binds to the Fe(II)-heme [15]. In fact, three paramagnetic forms of heme-nitrosylated Hb were observed in erythrocytes isolated from human and rodent blood, i.e. two forms of Hb α-chain; i) 5-coordinate (T-form, deoxy-Hb); and ii) 6-coordinate (R-form, oxy-Hb) NO complexes; and one form of Hb β-chain, 6-coordinate NO complex (R-form, oxy-Hb). Remarkably, the EPR spectrum of nitrosylated T-form (“HbNO” in the following text) was predominantly observed in venous blood and characterized by rhombic symmetry with a well-resolved triplet hyperfine structure (hfs) displayed at principal g_z_-value 2.01 (A_z_ = 16.8 G) due to net donation of electron density from Fe(II) to NO after cleavage of the bond between the heme iron and the proximal His residue of the R-form (reviewed in [14]). The nitrosylated T-form is less stable than the R-form with a half-life ~ 20 minutes [16], which emphasizes the interest to measure levels of nitrosylated T-form in venous blood as a dynamic marker of NO availability in the systemic circulation. The concentration of this HbNO complex as an index of NO availability was analyzed by EPR in animal [17, 18] and in human [19, 20] blood. We have previously developed an improved method of HbNO detection and found a correlation between the HbNO concentration and endothelial function assayed by digital microtonometry (ENDO-PAT) in a cohort of healthy volunteers [19]. However the respective contributions of NOS from the vessel wall or NOS within erythrocytes as predominant sources of heme nitrosylation remain undetermined, as are the factors influencing the stability of the HbNO complex formed in red blood cells (RBCs). In this study we determined the dependence of the HbNO complex formation on an erythrocytic NOS and its sensitivity to physico-chemical determinants such as partial pressure of oxygen (pO_2_), blood pH, and temperature. Our observations provide further understanding on the source of erythrocyte HbNO and identify optimal conditions for its stability *ex vivo* after isolation from venous blood.

## Materials and methods

### Blood collection from experimental animals and study subjects

Human blood was collected by a venopuncture from the median cubital vein into vacutainer tubes containing EDTA (K2E, Vacutainer, BD-Plymouth, UK) from healthy volunteers in the morning after a night fasting (n = 20). Procedures were approved by the local Ethics Committee of the Faculty of Medicine and Saint Luc university hospital of the Université Catholique de Louvain. All volunteers were individually informed about study procedures and signed a written informed consent.

Animal experiments were performed in eNOS knockout mice (eNOS^(-/-)^, n = 6) or caveolin-1 knockout mice (cav-1^(-/-)^, n = 4) and control littermates C57BL/6 (eNOS^(+/+)^, n = 14 or cav-1^(+/+)^, n = 4); and in Wistar rats (n = 20), all at 12–16 weeks old. After the anesthesia of animals (by intraperitoneal injection of ketamine/xylazine, 0.1/0.01 mg/g body weight) we collected venous blood by cardiopuncture (from the right ventricle), or retro-orbital puncture from mice, and/or venopuncture (jugular vein) from rats. In some experiments, a nitrovasodilator **(**1μmol/L of isosorbide dinitrate, I.V. diluted in saline solution) or vehicle were injected into the jugular vein of anesthetized rats before blood collection. Blood samples were collected every 5 minutes between 5 and 20 minutes after injection and were immediately frozen in calibrated tubes for EPR measurements at low temperature (77K). The procedures were approved by the Institutional Animal Care and Research Advisory Committee of the Université Catholique de Louvain.

### Isolation of venous erythrocytes

Erythrocytes were collected after blood centrifugation at 800-1000x*g* (10min. at 4°C) from the tube bottom and used immediately or after 3 washings with an isotonic buffer (in mmol/L: 150 NaCl, 5 D-glucose, 0.25 KH_2_PO_4_, 0.25 Na_2_HPO_4_, pH 7.4) followed by centrifugation at 800x*g* (5min.) where indicated.

### Calibration curve and HbNO quantification by EPR

**Fig 1:**
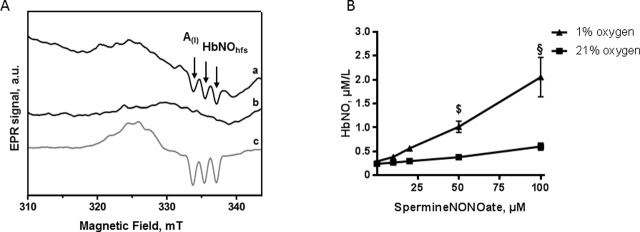
Effect of oxygen on HbNO (5-coordinate alpha-nitrosyl-hemoglobin) formation in human RBCs. A. Typical EPR spectra recorded in human erythrocyte samples exposed to 1% (a, c) or 21% (b) of O_2_ and treated with 50 μmol/L of Spermine-NONOate for 1 hour (a, b); or with a NO-generating mixture (4 μmol/L of NO_2_^-^ and 50 mmol/L of Na_2_S_2_O_4_) (c). Spectra were acquired as described in Materials and Methods. Arrows point to the triplet hyperfine structures (hfs). A(I) corresponds to the amplitude of the first hf component used for HbNO quantification. B. The concentrations of HbNO complex accumulated in human reconstituted RBCs (50% of haematocrit in isotonic buffer) under 21% (square) or 1% (triangle) of O_2_ after addition of graded concentrations of Spermine-NONOate. Data are shown as mean values ± SEM; ^$^ P < 0.01 ^§^P < 0.0001; n = 4 different RBC preparations.

For the detection of the 5-coordinate α-HbNO (HbNO) accumulated *in vivo* in RBCs we used low-temperature EPR spectroscopy as described previously (19). The HbNO concentration was determined from the EPR spectra using a calibration curve that was obtained from the HbNO EPR signals formed in samples of human or mouse erythrocytes as follows; erythrocytes were collected from venous blood, washed with isotonic solution followed by centrifugation (800x*g*, 10 min), then reconstituted at 50% of haematocrit, and incubated with a NO-donor system (sodium nitrite at 0.05–0.1–0.5–1–2–4 and 8 μmol/L and sodium dithionate, Na_2_S_2_O_4_, 50 mmol/L) in anaerobic condition (1% of O_2,_ 37°C; INVIVO_2_400 hypoxic chamber, Ruskinn Technology, Ltd, UK). Then the samples were frozen in calibrated tubes for low-temperature EPR measurements, and the EPR spectra were analysed using ESR MPlot&Analysis (Magnettech) or XENON (Bruker) software. The HbNO EPR spectrum displays the well-resolved triplet hyperfine structure (hfs) at g_z_ ~ 2.01 (S1 Fig). Linearity was observed between the increasing intensity of the HbNO EPR signals measured by double integration and the amplitude of the HbNO hfs components. For this reason the peak-to-peak amplitude of the hfs component was used for 5-coordinate α-HbNO quantification (Fig 1A) [19]. The real concentration of synthesized HbNO in these samples was quantified by comparison of the signal intensity obtained by spectra double integration with that of a common EPR standard (Cu–EDTA complex, 50 and 100 μmol/L frozen in 30% glycerol-water solution). The EPR spectra were recorded using either a Magnettech MiniScope (MS400) or a Bruker EMXmicro X-band EPR spectrometer with the following settings: microwave frequency ~ 9.35 GHz; modulation frequency, 100 kHz; microwave power (MW), 20 mW; modulation amplitude (MA), 7 G and 5 scans at 77K using an EPR quartz finger Dewar filled with liquid nitrogen.

### O_2_, T° and pH effects

To study HbNO accumulation upon incubation with a NO donor, RBCs were collected from venous blood, washed and reconstituted at 50% of haematocrit with isotonic buffer. Erythrocytes were incubated at 37°C during 1 hour in a hypoxia workstation (at 1% of O_2)_, or at room air (21% of O_2_). Then samples were incubated with a NO-donor ((Z)-1-[N-[3-aminopropyl]-N-[4-(3-aminopropylammonio)butyl]-amino]diazen-1-ium-1,2-diolate, Spermine-NONOate, Enzo Life Science) at different concentrations (10–20–50 and 100 μmol/L), during 1 hour and frozen in calibrated tubes in liquid nitrogen for low-temperature EPR measurements.

To study factors influencing HbNO stability, we first allowed the accumulation of HbNO complexes in human blood immediately after collection by addition of 100 μmol/L of Spermine-NONOate directly into tubes protected from room air (using a syringe). Blood was incubated at venous O_2_ level during 45 minutes, centrifuged, then the tube was opened at 21% or at 1% of O_2_ and RBCs samples were frozen every 10 min for EPR measurements. The influence of pH was measured in RBCs reconstituted at 50% haematocrit with PIPES buffer (P2949, Sigma; monosodium salt) at different pH (6.2, 7.2, 7.4), or with acetic acid (pH 4.7). The influence of temperature was measured by incubating reconstituted RBCs at 37°C or 20°C. Erythrocyte samples were frozen every 10 minutes until 50 minutes in calibrated tubes for EPR measurements.

### Blood gas analysis

Blood oxygenation and percentage of oxy- and deoxy-hemoglobin were analysed by a blood gas analyser (ABL725, Radiometer, Copenhagen, Denmark) in additional blood samples collected and incubated in parallel in the same conditions from the same volunteers.

### Human and murine erythrocyte ghost membranes isolation

Washed erythrocytes were re-suspended in cold lysis buffer (11 mmol/L Tris-HCl, 1 mmol/L PMSF pH7.6) at 1:10 (v:v) ratio and incubated on ice for 30 minutes; cell membranes were collected by ultracentrifugation (25,000x*g* for 20 minutes at 4°C) followed by several washes until a pale/pinkish pellet was observed. Mouse and human RBCs, membrane ghosts, mouse lung and human aortic endothelial cells were lysed in RIPA buffer (50 mmol/L Tris, 150 mmol/L NaCl, 1% Triton-X-100, 0.05% sodium deoxycholate, 1 mmol/L EDTA, 0.1% SDS, pH 7.4) with protease inhibitor cocktail (P8340, Sigma), sonicated and centrifuged at 10,000x*g* for 10 minutes to remove cell debris. Total proteins concentrations were determined by the Bradford assay (5000006, BioRad).

### Immunoprecipitation and western blot analysis

Immunoprecipitation was performed using Dynabeads protein G (1003D, ThermoFisher) according to manufacturer’s protocol. Briefly, 500μg of protein samples were incubated overnight at 4°C under agitation with magnetic beads coupled to 5μg of monoclonal mouse eNOS antibody (610297, BD Biosciences). After several washes with washing buffer (Invitrogen), immunoprecipitated proteins were eluted with elution buffer provided by the manufacturer and processed for western blotting. For western blot analysis, denatured proteins were loaded and separated on 10% or 12.5% SDS polyacrylamide gel (Bio-Rad) and transferred to PVDF membranes. Membranes were blocked with 1% nonfat dry milk in Tris-buffer saline solution with 0.1% Tween 20 (TBST, Sigma), probed overnight at 4°C with eNOS antibody (1:500) or monoclonal caveolin-1 antibody (1:500) (610407, BD Biosciences), washed in TBST and then incubated for 1 hour with goat anti-mouse antibody (1:5000, Jackson). Bands were detected using a chemiluminescent reagent (ECL) by an Amersham Imager 600 (GE Healthcare Life Sciences).

### Flow cytometry analysis

40μl of washed human and rat RBCs (5x10^8^ cells) were fixed overnight in 1mL of paraformaldehyde 4% at 4°C. For permeabilization, after washing with staining buffer (PBS with 1% FBS, 0.09% NaN_3_), the cell pellet was suspended in 0.5mL of 0.1% Triton for 10 minutes. After one wash to remove Triton X-100, cells were incubated in FBS 10% for 15 minutes at 4°C. Erythrocytes were incubated with 2.5μg of mouse anti-eNOS (610297, BD Biosciences) (1 hour) and 1μg of polyclonal rabbit anti-Aquaporin-1 (AB2219, Millipore) (20 minutes) antibodies at room temperature (RT). The secondary antibodies Alexa-647 goat anti-mouse and Alexa-488 goat anti-rabbit (Invitrogen) were incubated for 20 minutes at RT. Negative controls were obtained by incubating only with secondary antibodies and with mouse non-specific IgG1,k Alexa-647-conjugated (Invitrogen). Finally, RBCs were suspended in staining buffer. Data acquisition was performed with a FACSCantoII™ flow cytometer (BD Biosciences) and data analysis with FlowJo software (BD Biosciences).

### Immunofluorescence microscopy

Freshly isolated human RBCs were smeared across glass slides and allowed to air-dry for 15 minutes, then were fixed in 4% paraformaldehyde at room temperature for 30 minutes. After extensive washing, they were incubated for 10 minutes with 0.1% TritonX-100 for permeabilization. RBCs were immunolabeled with anti-eNOS and anti-aquaporin-1 antibodies for all night, followed by washing and incubation with anti-mouse or anti-rabbit Alexa Fluor-conjugated secondary antibodies for 1 hour. The preparations were mounted in fluorescent mounting medium (DAKO) and examined in a fluorescence microscope equipped with an ApoTome module for structured illumination (Zeiss AxioImager.z1, USA).

### Measurements of eNOS activity *ex vivo* in mouse erythrocytes by EPR

Freshly isolated RBCs from eNOS^(+/+)^ and eNOS^(-/-)^ mice were first incubated for 1 hour at room air, in order to decrease the HbNO complexes formed *in vivo*, then were treated with L-NAME (Nω-Nitro-L-arginine methyl ester hydrochloride; N5751, Sigma; 30 mmol/L in Krebs-Henseleit buffer, KHS) or nor-NOHA (N-ω-Hydroxy-nor-L-arginine; F-3685, Bachem; 10 mmol/L in KHS) or with both or with vehicle (KHS). After 10 minutes of incubation, 3 mmol/L of L-Arginine (A-3784, Sigma) was added to each sample and RBCs were incubated for 30 minutes at 37°C. Samples were then frozen in liquid nitrogen in calibrated tubes for low temperature EPR measurements. In order to quantify the triplet hyperfine structure of the HbNO EPR signal, the overlapping EPR signal of protein-centered free radicals (PFR) was subtracted. A prominent PFR EPR signal was observed in plasma samples of mice treated with L-NAME (1 g/500 mL in drinking water, during 5 days).This PFR EPR signal had a line shape similar to the PFR signal observed in mouse erythrocytes samples; and was used for digital subtraction after division by a coefficient calculated from the ratio of its intensity to the intensity of corresponding signal of interest using Xenon software as described in [19]. The resulting difference spectrum revealed the unmasked triplet hfs of HbNO that was used for the quantification. Low-temperature EPR spectra were recorded, using a quartz finger Dewar filled with liquid nitrogen at 77 K, on a Bruker EMXmicro X-band EPR spectrometer with the following setting: microwave frequency ∼ 9.35 GHz; modulation frequency, 100 kHz; MW, 20 mW; MA, 7 G; 10 scans.

### Measurements of erythrocyte eNOS activity *ex vivo* by nitrite and nitrate colorimetric assay

Mouse blood samples used for the EPR analyses were stored at 20–25°C in the dark until subsequent thawing for nitrite/nitrate assay. Human RBCs were collected as described above and used for NO_2_^-^ and NO_3_^-^ quantification immediately. Briefly, after hemoglobin precipitation with cold ethanol/chloroform (v: v– 1: 5) and vortex agitation, the mixture was centrifuged at 10,000x*g* for 10 minutes at 4°C. Aliquots of clear supernatants were used to detect NO_2_^-^ and NO_3_^-^. For nitrite measurements, supernatants were incubated with Griess reagents according to Griess Reagent Kit instructions (G-7921, ThermoFisher). The nitrite concentrations were determined using a calibration curve (1–100 μmol/L) of sodium nitrite in deionized water. For nitrate measurements, NO_3_^-^ was first reduced to NO_2_^-^ in presence of NADPH 80 μmol/L (N-7505, Sigma) and nitrate reductase 50mU / 100 μl (N-7205, Sigma). The resulting total concentrations of nitrite and nitrate in samples (including products formed after NO oxidation) were determined by comparison with a calibration curve (1–100 μmol/L of sodium nitrite in deionized water). The nitrate concentrations were determined by the difference between the total NO oxidation products and nitrite concentrations from the same aliquots. Measurements of the absorbance at 548 nm were performed with a Spectramax i3 (Molecular Devices, LLS, USA).

### Measurement of erythrocyte eNOS influence on HbNO decay *ex vivo* by EPR

Vehicle or 30 mmol/L L-NAME or 10 mmol/L nor-NOHA and 3 mmol/L L-Arginine were added to erythrocytes isolated from mouse or rat blood as described above; human venous blood was incubated with anti-oxidants (ascorbic acid, 5 mmol/L and N-acetyl-cysteine, 5mmol/L) (A4544, Sigma and A8199, Sigma) to reduce the formation of redox-sensitive PFR in erythrocytes and with 30 mmol/L L-NAME, or 10 mmol/L nor-NOHA, or 3 mmol/L L-Arginine; after centrifugation, plasma was conserved in vacutainer tubes in order to preserve the HbNO. RBCs were incubated at 21% of O_2_ and at 37°C before collection at 0, 5, 20 and 40 min and frozen for EPR measurements. The HbNO EPR spectra from human blood samples were quantified as described before [19]. Briefly, the Protein Free Radicals (PFR) EPR signal for digital subtraction was generated as difference spectrum between two EPR spectra from two RBC samples that were prepared from the blood of the same human subject; one was exposed to open air in the presence of an antioxidant mixture (ascorbic acid and N-acetyl-cysteine, 5 and 5 mmol/L), for 45 minutes; the other was similarly incubated at open air, but without antioxidants. The differential PFR signal was subtracted from the whole EPR spectra for quantification of the unmasked HbNO. All spectra were recorded at low temperature (77K) using a Bruker EMXmicro X-band EPR spectrometer as described above; subtraction and analysis were performed with Xenon software.

### *In vivo* administration of L-NAME or a NO donor

Mice and rats received tap water containing distilled water or L-NAME (1 g/L) from day 0 to day 7. To evaluate the blood HbNO levels we anesthetized rats and mice with isoflurane inhalation and collected venous blood by retro-orbital puncture on day 1 and 7. NO donor (1 μmol/L of isosorbide dinitrate, Cedocard^R^ I.V. 0.1%) or vehicle was administered into the jugular vein as described above. Blood samples were collected and immediately frozen in calibrated tubes for EPR measurements.

### Erythrocyte HbNO measurements in cav-1^(+/+)^ and cav-1^(-/-)^ mice

Cav-1^(+/+)^ and cav-1^(-/-)^ mice [17, 21] were anesthetized and venous blood was collected by retro-orbital puncture. Blood samples were centrifuged and isolated RBCs were immediately frozen in calibrated tubes for low-temperature EPR measurements, as described above.

### Statistics

Results are shown as mean values ± SD, or as mean values ± SEM. To analyze the differences in nitrite/nitrate and HbNO productions in murine samples, a Kruskal-Wallis with Dunn’s test (for multiple comparisons) was performed. One-way ANOVA followed by a Tuckey’s test was performed once normality had been verified by Shapiro–Wilks test, for nitrite/nitrate measurements in human samples. The effects of L-NAME in drinking water on HbNO *in vivo* formation in mice and rat and the difference between the basal HbNO level of cav-1^(+/+)^ and cav-1^(-/-)^ were analyzed with Mann-Whitney test. All the others differences in human and rat erythrocytes (treated or untreated) were analyzed by Two-way ANOVA followed by Sidak’s test. All reported *P*-values were two sided and were considered statistically significant at *P* < 0.05.

## Results

### 1. Formation and stability of HbNO in isolated erythrocytes under different oxygen levels

Upon incubation of human RBCs with NO-donor (Spermine-NONOate) during 1 hour under 1% of O_2_, we observed the formation of the typical EPR spectrum of HbNO with triplet hfs corresponding to 5-coordinate nitrosyl-hemoglobin (T-form) (spectrum a in Fig 1A). Spectra corresponding to nitrosyl-Hb in the R form were also observed under 21% of O_2_ (spectrum b in Fig 1A). The accumulation of HbNO (T-form) was less prominent at 21% compared with 1% of O_2_ (Fig 1B; 0.0036 ± 0.0004 μmol HbNO/μmol NO-donor versus 0.018 ± 0.002 μmol HbNO/μmol NO-donor formed at 1% O_2;_ P<0.03).

**Fig 2:**
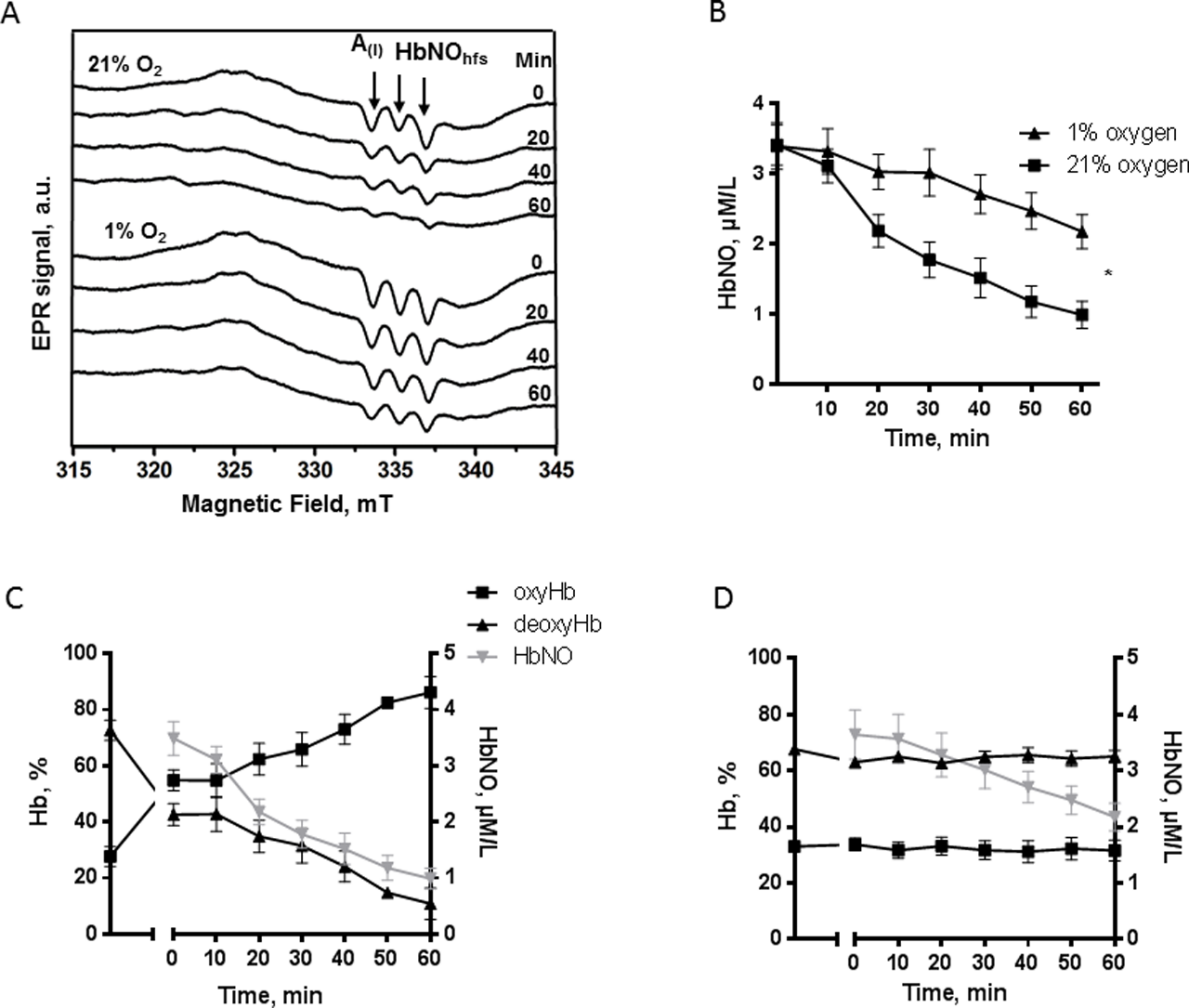
Effect of oxygen on stability of HbNO formed *ex vivo*. A. Typical EPR spectra, and B. concentrations of HbNO complex accumulated in human RBCs pre-incubated with Spermine-NONOate (100 μmol/L, for 60 minutes, under venous O_2_ level) and subsequently exposed for 60 minutes to 21% (square) or 1% (triangle) of O_2_ as described in Methods. Arrows in (A) point to the triplet hyperfine structures (hfs); A(I) indicates the amplitude of the first hf component. Data in B are shown as mean values ± SEM; ^*^ P < 0.05; n = 4 different RBC preparations. C-D. Correlation between HbNO concentrations in RBCs and percentage of oxy/deoxy hemoglobin in venous blood during incubation at 21% (C); and 1% of O_2_ (D).

We next tested the influence of erythrocyte oxygenation on the stability of HbNO pre-formed in RBCs under venous pO_2_ level. Typical EPR spectra and calculated HbNO concentrations are shown in Fig 2A and 2B. Low oxygen level preserved the HbNO stability; 12.0 ± 0.2% of HbNO content was lost after 30 minutes at 1% O_2_, compared to 47.7 ± 0.2% under 21% O_2_ (Fig 2B). Aliquots of human blood were collected in parallel from the same volunteers and the percentage of oxy- and deoxy-hemoglobin was determined by blood gas analyser after exposure to 1% or 21% O_2_. During incubation at 21% O_2_ the oxy-Hb formation increased up to 90% while deoxy-Hb decreased to 10% (Fig 2C), whereas at 1% O_2_ the oxy- and deoxy-Hb remained at their original levels (Fig 2D). Notably, the concentration of HbNO declined much more quickly under 21% O_2_, as expected, and was correlated with the decreased content of deoxy-Hb (Fig 2C); conversely, HbNO concentrations were better conserved under 1% O_2_, which maintained stable levels of deoxy-Hb (Fig 2A bottom spectra and 2D). To verify whether nitrosylated Hb in R form would appear under re-oxygenation, we digitally subtracted the model spectrum HbNO (5-coordinate Fe(II)NO at α-chain Hb) from the experimental EPR spectra presented in Fig 2A (upper spectra). The spectra were normalized before subtraction to minimize residual signal of HbNO (5-coordinate Fe(II)-NO at α-chain) and the resulting spectra were compared with a model signal of nitrosylated Hb in R-form, obtained in oxygenated RBCs. The calculated intensity of residual signal (heme–iron–NO complex in a six-coordinate geometry, R form of Hb) was less than 28% initially and did not increase with time upon oxygenation up to 60 min (S2A Fig). Furthermore, the stability of HbNO was preserved at room air if a column of plasma was left above the RBC after centrifugation to slow down the diffusion of O_2_ into erythrocytes (S2B Fig).

### Effect of temperature and pH on HbNO stability

**Fig 3:**
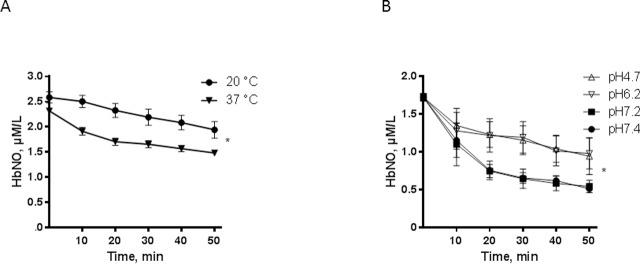
Temperature and pH influence on HbNO stability. A. Stability of HbNO complex accumulated in human RBCs pre-incubated with Spermine-NONOate (100 μmol/L, 60 minutes) under venous O_2_ level, and subsequently incubated at 20°C (circle) or 37°C (triangle) during 50 minutes under 21% of O_2_. B. Effect of pH on HbNO stability in human RBCs pre-incubated with NO-donor and reconstituted at 50% haematocrit with isotonic buffer at acidic pH (6.2–4.7) or at physiological pH (7.4–7.2) at 21% of O_2_ and 37°C. Data are shown as mean values ± SEM; * P < 0.05; n = 4 different RBC preparations.

Next, we determined the effect of temperature on the stability of the HbNO complex by incubating human venous RBCs at 20°C or 37°C. HbNO was more stable at 20°C compared to 37°C (Fig 3A); a decrease of HbNO by 15.2 ± 0.3% was observed at 20°C compared to 29.6 ± 0.1% at 37°C after 30 minutes of RBC incubation. Incubation at acidic pH (6.2–4.7) also resulted in better preservation of HbNO compared to near physiological pH (7.4–7.2) at 21% of O_2_: HbNO was decreased by 31.7 ± 0.8% at 30 min compared to 62.2 ± 0.4% at physiological pH (Fig 3B); a less pronounced but significant increase of HbNO stability was also observed at the acidic pH (6.2–4.7) under 1% of oxygen concentration (S3 Fig).

### 2. Expression and activity of eNOS in rodent and human erythrocytes

**Fig 4:**
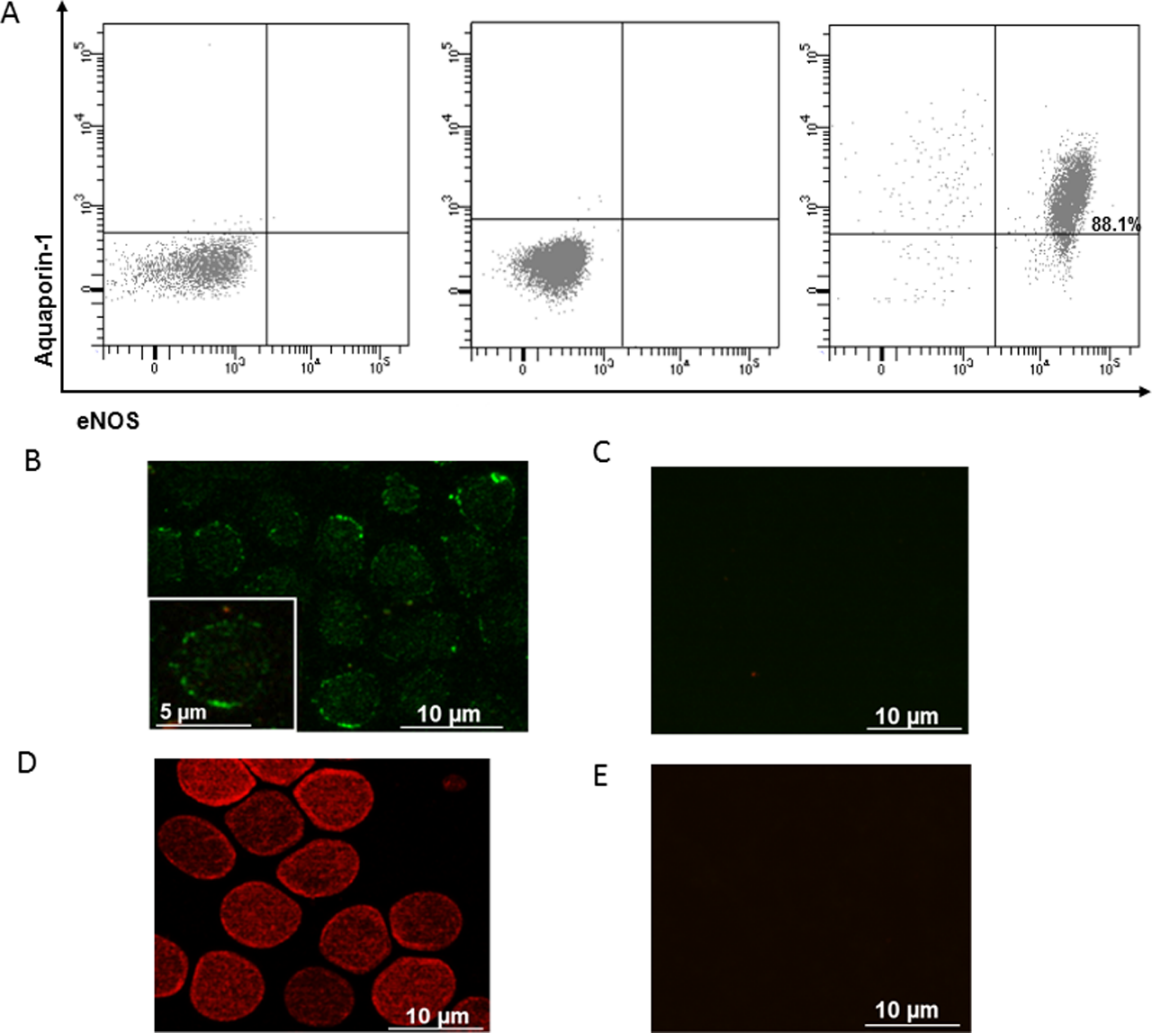
Human RBCs express eNOS proteins. A. Representative flow cytometric two parameter dot plot of isolated RBCs from a healthy subject co-stained with primary anti-Aquaporin-1 and secondary Alexa Fluor-488-conjugated anti-IgG antibodies; and primary anti-eNOS and secondary Alexa Fluor-647-conjugated anti-IgG antibodies. Panel on the left shows co-staining only with conjugated secondary antibodies, panel in the middle shows staining only with mouse isotype IgG1,k Alexa-647-conjugated (negative controls). Panel on the right shows the identification of RBCs as eNOS–positive and Aquaporin1-positive events in the upper right quadrant. The percentage of RBCs double positive is indicated in the upper right quadrant. (B-E) Representative images of eNOS (B) and AQP1 (D) detection in healthy human RBCs using immunofluorescence microscopy. As negative controls, RBCs were stained only with conjugated secondary antibodies (Alexa Fluor-488 anti-mouse IgG for eNOS (C) and Alexa Fluor-568 anti-rabbit IgG for AQP1 (D)).

To test for the presence of the eNOS isoform in erythrocytes, we analyzed human RBCs by flow cytometry and immunofluorescence microscopy, and performed western blotting analysis on RBC samples from eNOS^(-/-)^ mice and their wild-type littermate. Fig 4 illustrates the results of flow cytometry on human erythrocytes double-stained with antibodies for eNOS and aquaporin-1 (AQP1), one of the most abundant proteins in RBCs. The majority (88%) of the erythrocytes population are double positive for eNOS and aquaporin 1 showing a shift from the bottom-left quadrant, when they are stained only with secondary antibodies, to the upper-right quadrant of the panel (Fig 4A). We also observed eNOS and AQP1 expression in human erythrocytes using immunofluorescent microscopy (Fig 4B and 4D).

Moreover western blotting showed a signal for eNOS in whole lysates and membrane extracts of RBC from eNOS^(+/+)^ mice as well as human RBC, while no signal was observed in RBC from eNOS^(-/-)^ mice (S4A and S4B Fig).

**Fig 5:**
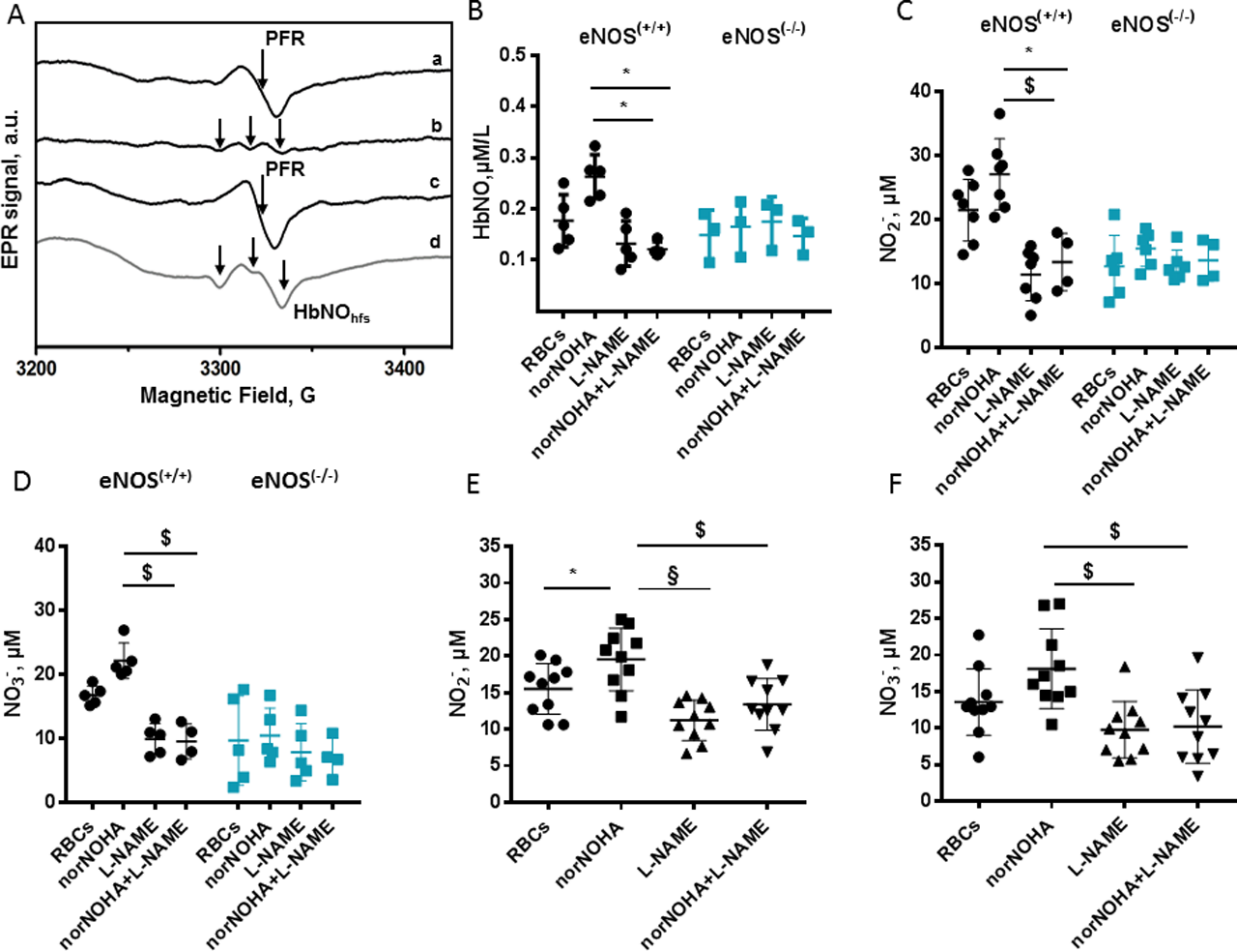
Detection of erythrocyte eNOS activity by EPR spectroscopy and by nitrite and nitrate colorimetric assay. A. Typical procedure used for subtraction analysis: the EPR spectrum of RBC sample before (a) and after (b) subtraction of PFR EPR spectrum (c) obtained as described in Material and Methods. Typical HbNO EPR signal from immediately frozen mouse whole blood (d); the hyperfine structure (hfs) of HbNO is shown by the arrows. (B-F). Quantification of erythrocyte HbNO EPR signals (B) and products of NO oxidation, nitrite and nitrate formed in isolated RBCs of eNOS^(+/+)^and eNOS^(-/-)^ mice (C and D) and in human erythrocytes (E and F), after incubation *ex vivo* with norNOHA and/or L-NAME as described in Materials and Methods. Data are shown as mean values ± SD; *P < 0.05, ^$^P<0.01, ^§^ P < 0.0001; n = 3–7 different mouse RBC preparations and n = 10 different human preparations.

We next determined the activity of eNOS in human and mouse RBCs from eNOS^(+/+)^ and eNOS^(-/-)^genotypes. To test the impact of arginase-1, an enzyme consuming L-arginine, a common substrate with eNOS, we monitored NOS activity in RBCs incubated *ex vivo* with L-arginine with or without the arginase-1 inhibitor, Nor-NOHA, in parallel with the NOS inhibitor, L-NAME. NO production was assessed in isolated RBCs by measuring the accumulation of the NO oxidation products (nitrite and nitrate) and HbNO formation (by EPR). Note that for these experiments, RBC were first exposed for 1 hour at room air to allow the decay of HbNO complexes accumulated *in vivo*. A subtraction method was used to unmask the hfs typical signal for HbNO overlapping with the PFR signal (as described in Materials and Methods and illustrated in Fig 5A). None of these treatments had any effect on HbNO in RBCs from eNOS^(-/-)^ mice. However, in RBCs from eNOS^(+/+)^ mice, we observed an increase of HbNO after arginase-1 inhibition, which was abrogated upon NOS inhibition (Fig 5B). Similar results were obtained for nitrite and nitrate accumulation in RBCs. L-NAME with or without nor-NOHA had no effect on nitrite or nitrate accumulation in RBCs from eNOS^(-/-)^ mice, whereas nor-NOHA treatment increased nitrite and nitrate accumulation in RBCs from eNOS^(+/+)^ mice; this increase was abrogated upon NOS inhibition with L-NAME (Fig 5C and 5D). Notably, similar observations were obtained with RBCs from human volunteers, with an increase of nitrite and nitrate upon incubation with nor-NOHA that was inhibited by L-NAME (Fig 5E and 5F).

### Contribution of erythrocyte eNOS to the HbNO content

**Fig 6:**
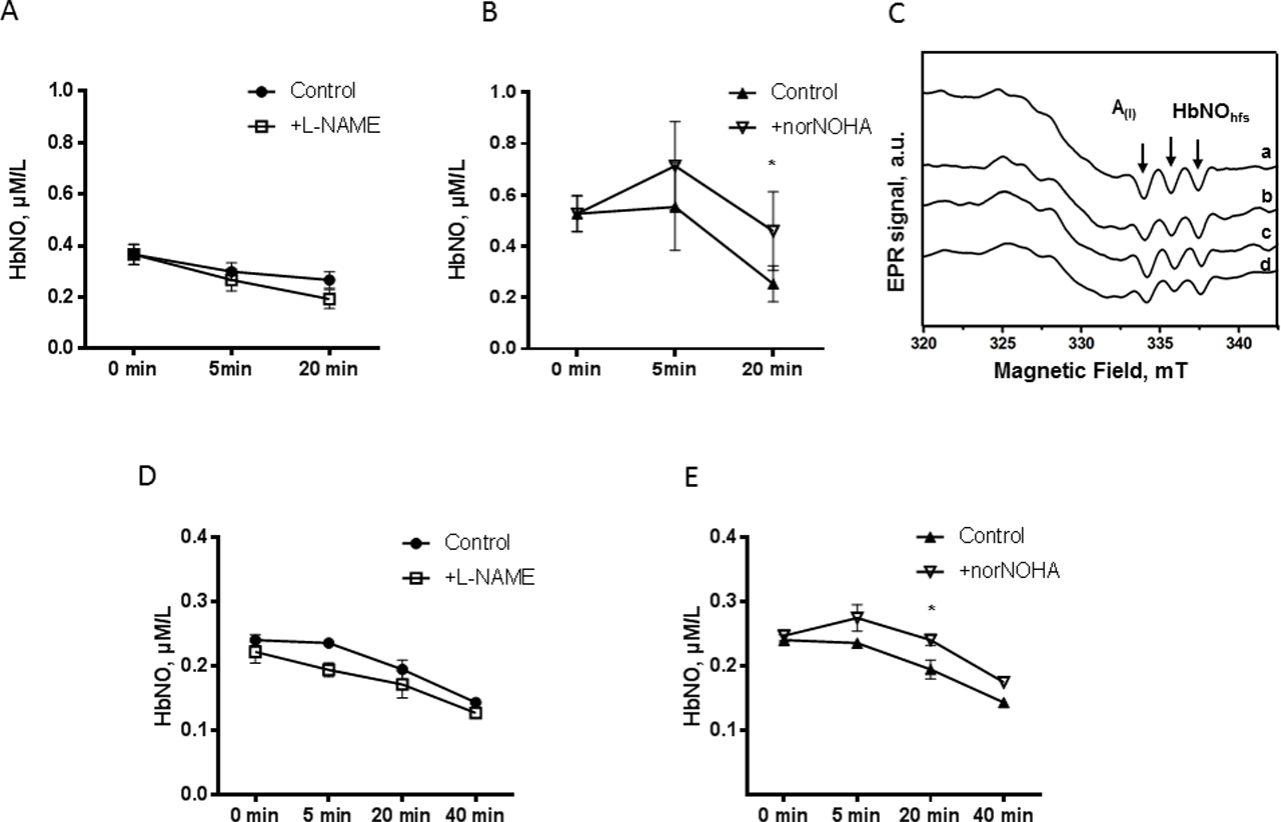
Minimal influence of erythrocyte eNOS activity on HbNO content. Time-dependent decay of EPR HbNO signal formed *in vivo* in mouse (A and B) and in human (D and E) erythrocytes after *ex vivo* treatment with L-NAME or vehicle (A and D) or with nor-NOHA or vehicle (B and E) under L-Arginine supplementation at 21% of O_2_ as described in Materials and Methods. C. Typical EPR spectra recorded in mouse erythrocyte samples before (a) and after incubation with L-Arginine alone (b) or with nor-NOHA (c) or L-NAME (d) for 20 minutes at 21% of O_2_. Sample aliquots were frozen at 0, 5, 20 and 40 minutes. The EPR spectra were acquired as described in Material and Methods. Data are shown as mean values ± SEM; n = 3 different RBC preparations. * *P* <0.05 at 20 min.

Having identified an active NOS in RBCs, we next determined its contribution to HbNO content measured in freshly isolated RBCs, which theoretically integrated erythrocyte NO production as well as NO produced from the surrounding vascular wall *in vivo*. We monitored the decay of HbNO formed *in vivo* in freshly isolated mouse (Fig 6A and 6B) and human (Fig 6D and 6E) RBCs, upon addition of L-NAME or nor-NOHA at 21% of O_2_ *ex vivo* as described in Materials and Methods. nor-NOHA produced a slight but significant increase of HbNO accumulation at 20 min (Fig 6B and 6E); but L-NAME had no significant effect on HbNO decay (Fig 6A and 6D). Typical EPR spectra obtained from the mouse RBC samples are shown in Fig 6C. We repeated the experiment in rat erythrocytes that typically exhibit larger HbNO content and also express eNOS (as verified by us) (S4C Fig). Rat RBCs were treated *ex vivo* with nor-NOHA and/or L-NAME at 4% or 21% of O_2_. Under 4% of O_2_, L-NAME had no effect on the slow decay of HbNO (S5A Fig). Under 21% of O_2_, L-NAME produced an early decay of HbNO signal at 5 min but the effect was not sustained at later time points (S5B Fig). Likewise, nor-NOHA had no measurable effect on HbNO content of RBC incubated at 21% of O_2_ (S5C Fig).

### Comparative erythrocyte HbNO content upon systemic NOS inhibition, caveolin-1 genetic deletion or infusion of a NO donor *in vivo*

**Fig 7:**
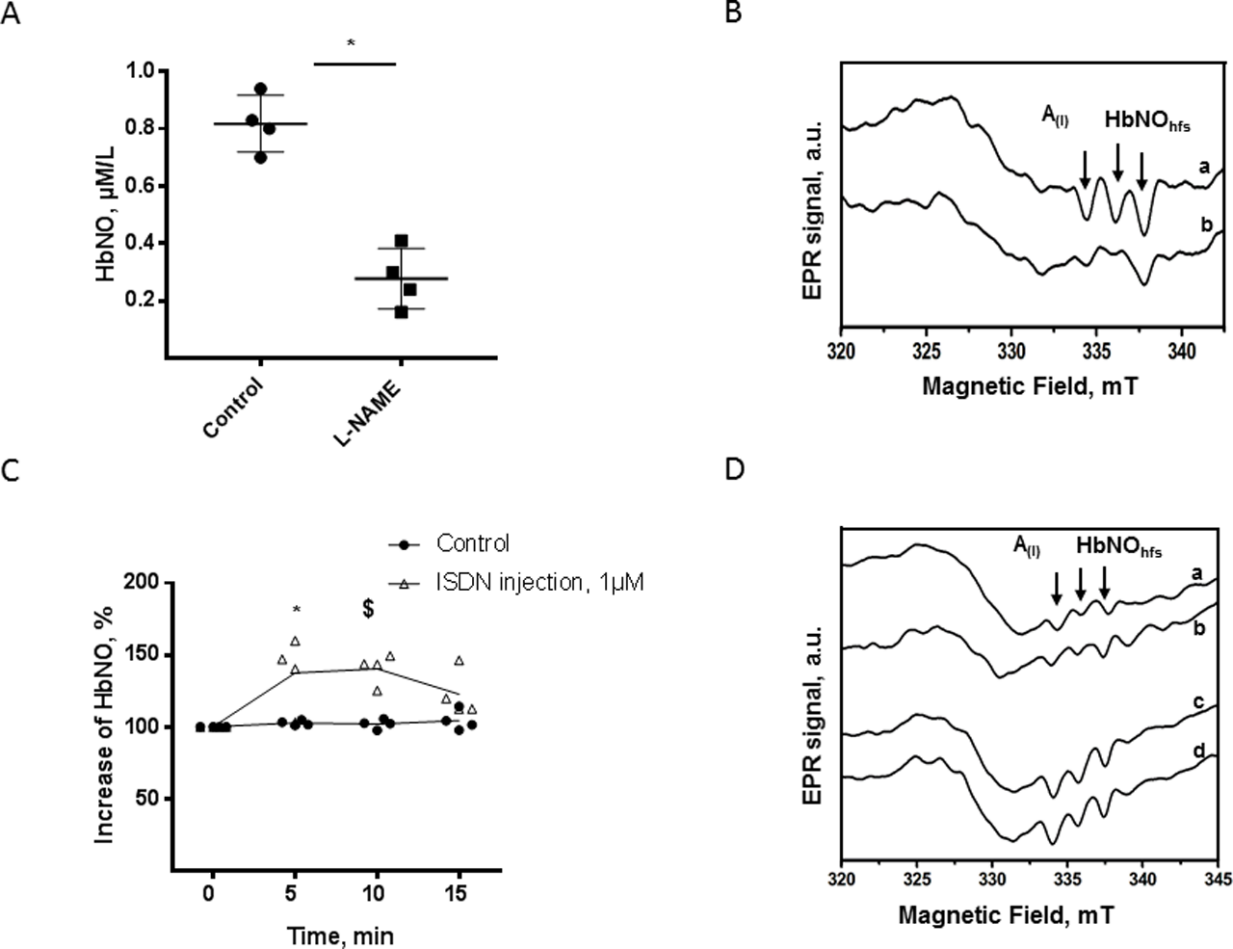
Sensitivity of blood HbNO formation to *in vivo* administered L-NAME or a nitrovasodilator. A. Concentration of HbNO in venous mouse RBCs after seven days of treatment with L-NAME or vehicle as described in Materials and Methods. B. Typical EPR spectra of HbNO recorded from isolated RBCs after one week administration of vehicle (a) or L-NAME (b) in drinking water. C. Kinetics of relative *in vivo* changes of HbNO EPR signal in venous rat blood after *in vivo* isosorbide dinitrate (ISDN) or vehicle injection. D. Typical HbNO EPR spectra after 5 and 10 minutes i.v. administration of vehicle (a-b) or isosorbide dinitrate (ISDN) (c-d). Data are shown as mean values ± SD; ^*^ P < 0.05 and ^$^ P < 0.01; n = 4 different RBC preparations.

Our observations above suggest that the HbNO content measured in freshly drawn RBCs predominantly originates from paracrine and/or systemic sources. To test this, we first compared the HbNO content of RBC freshly drawn from mice and rats treated/or not with L-NAME in the drinking water for 7 days. As shown in Fig 7A and 7B, a stronger decrease of about 65%, was observed in HbNO signal from mouse RBCs after one week of L-NAME administration in drinking water, compared to RBC from untreated, control mice. In rat RBCs, L-NAME treatment *in vivo* progressively reduced the HbNO content (S6 Fig).

Next, we tested an intervention that would modulate eNOS specifically in vascular endothelial cells. As we and others have shown that caveolin-1 allosterically inhibits eNOS, we first examined whether caveolin-1 is expressed in erythrocytes, by comparison with its well-established expression in endothelial cells. We found no expression of caveolin-1 in mouse erythrocytes, neither in membrane nor in cytosolic fractions, whereas a clear immunoblotted signal was observed in lung extracts (with rich endothelial content; S7A and S7B Fig). Therefore, genetic caveolin-1 deletion would only affect the activity of the vascular eNOS, not eNOS in erythrocytes. We then compared the HbNO concentration in erythrocytes freshly drawn from cav-1^(+/+)^ or ^(-/-)^ and found a clear increase (70%) in HbNO in erythrocytes from cav-1^(-/-)^ mice compared with cav-1^(+/+)^ (S7C and S7D Fig.). These data support a prominent influence of vascular endothelial eNOS on the HbNO content of circulating erythrocytes.

Finally, we injected rats with the NO donor, isosorbide dinitrate (1 μmol/L) in the jugular vein and serially collected venous blood every 5 minutes to measure the kinetics of HbNO formation in erythrocytes after exposure to systemic NO. Equal volumes of solvent were injected in control rats in parallel. As shown in Fig 7C and 7D, HbNO remained unchanged in control, solvent-injected rats. Conversely, a single injection of isosorbide dinitrate induced about 40% increase in RBC HbNO content after the first 5–10 min post-injection.

## Discussion

In the present study, we characterized the biochemical parameters that influence the formation and stability of heme (FeII)-nitrosylated hemoglobin (5-coordinate heme Fe(II)-NO at the α subunit of hemoglobin) in intact freshly-isolated erythrocytes; we also detected eNOS in rodent and human erythrocytes and tested if modulation of vascular and erythrocyte NOS activity *in vivo* or *ex vivo* influence the HbNO formation or stability in these models.

Dysregulation of NO production and availability in the circulation promotes the development of endothelial dysfunction and aggravates the severity of cardiovascular diseases. NO availability is dependent on intracellular and extracellular factors: NO production is mainly regulated by eNOS activity in the endothelium and by NO reactivity with different target molecules such as heme proteins (soluble guanylate cyclase and hemoglobin), thiol-containing molecules or “off-target” NO oxidation [22]. Its high reactivity rate explains the short half-life *in vivo*, thereby limiting strategies for its detection to the measurement of its reactions products or downstream messengers. These include the measurement of NO oxidation products (nitrite, nitrate and whole NO_x_) in plasma and tissues, cGMP in plasma and urine, as well as stable isotope methods for quantification of L-arginine conversion to L-citrulline in animal and clinical studies [7, 23]. However these assays do not accurately represent NO bioavailability and production rates. The NO_x_ level can be affected by dietary NO_x_ supplementation, metabolism and excretion by the kidney [24, 25], cGMP formation is altered by NO-independent activation of guanylate cyclase [26] and different studies using stable isotopic methods show limitations of specificity and sensitivity [23]. On the contrary, NO bound to hemoglobin in erythrocytes forms a relatively stable iron-nitrosyl complex (5-coordinate-α-HbNO in this study) measurable quantitatively by EPR in mouse, rat and human venous blood *ex vivo* [27]. Several groups detected the formation of HbNO in rodent blood *in vivo* and proposed it as a surrogate index for NO-modulated functions in the circulation [17, 18]. In human blood, however, HbNO was detected only under NO gas inhalation by patients [14, 28, 29]. Basal levels of HbNO formed *in vivo* were considered too low to be detected by EPR with a lower threshold limit of 200 nmol/L (reviewed in [27]). Analytical methods using ozone-based chemiluminescent detection showed residual HbNO levels of about 20 nmol/L after extensive procedure for hemoglobin isolation from blood [11]. Our group developed an assay to analyze HbNO concentrations in intact, snap-frozen erythrocytes, using an EPR subtraction method that allowed to resolve and quantify HbNO hfs masked by overlapping PFR and ceruloplasmin EPR signals (described previously in [19]). Using this approach we previously found a correlation between HbNO content in erythrocytes and endothelial function (independently measured by microtonometry) as well as cardiovascular risk factors [19, 20]. However, the analysis of the stability of HbNO formed in intact erythrocytes under biochemical conditions reflecting the vascular milieu were never taken into account in previous studies. Multiple biological factors such as compartmentation of Hb in the cytosol/membrane, proximity with oxidant/antioxidant proteins, local pH and physiological levels of oxygen do influence NO reactivity in intact erythrocytes compared to cell-free hemoglobin. To resolve this, we examined the stability of HbNO in erythrocytes isolated from freshly drawn venous blood of human volunteers and rodents under different conditions. We observed the predominant formation of 5-coordinate α-HbNO at venous levels of oxygen saturation (~ 35 ± 3 mm Hg), corresponding to 1–4% of O_2_ in hypoxia chamber, under incubation with exogenous NO-donor. Conversely, saturation of RBCs with oxygen (to 21%) increased NO dissociation without increasing the formation of EPR signals corresponding to 6-coordinate HbNO (at α- or β-subunit of Hb in R-form) (Figs 2A and S2A). The 6-coordinate HbNO at α and β-chains forms were previously observed in human and rat arterial blood under NO-gas or NO-donor administration *in vivo*. An effect of T-to-R transition under oxygenation on 6-coordinate HbNO formation was undetectable in our experimental conditions. This may be due to the influence of the NO bond at α-subunit of Hb on oxygen dissociation from the other heme sites, as was hypothesized before (reviewed in [30, 31]). The oxidation of HbNO (in T-form) with the ensuing conversion of NO to NO_x_^-^ could also explain the undetectable transformation to 6-coordinate HbNO [32]. We found that the half-life of HbNO in erythrocytes was about 60 minutes at low oxygen saturation (< 4%), surprisingly larger than *in vitro* NO dissociation from free hemoglobin [16, 28]. This could be explained by stabilizing effects of factors in the intracellular milieu, subcellular hemoglobin localization, and/or following NO re-capture by Hb [33]. Indeed, we observed stabilization of HbNO (5-coordinate α-HbNO) after erythrocytes incubation in a buffer at acidic pH (< 6.2), in line with the known sensitivity of hemoglobin Fe-proximal-His bond to allosteric effectors such as protons (Bohr effect), which would promote the dissociation of oxygen from hemoglobin (in line with previous observations [31]) and preserve the HbNO complex in normoxic condition. We also observed an increase of HbNO stability at lower temperature compared to 37°C in isolated erythrocytes *ex vivo*. This could be due to a reduction of temperature-sensitive oxidative reactions, thereby slowing down the HbNO decay. Indeed, reduced thiols are reciprocally decreased by hyperthermia (41.5°C) in many tissues such as liver and intestinal mucosa, with a powerful protective effect of *N*-acetylcysteine, an antioxidant known primarily as a thiol precursor and a substrate for GSH synthesis, even at 37°C [34, 35].

We further examined the origin of NO accumulated as HbNO in erythrocytes *in vivo*. As previous studies showed expression and activity of an eNOS-like enzyme in human RBCs [7–9] which could compete with arginase-1 for their common substrate L-arginine [9], we first confirmed the expression of eNOS in RBCs by flow cytometry analysis (Fig 4A), immunofluorescence microscopy and western blot analysis, in which specificity of immunodetected signals was verified by their absence in RBC extracts from eNOS^-/-^ mice (using an antibody known to recognize both mouse and human eNOS) (S3 Fig). Although the eNOS-like enzyme was previously demonstrated to be catalytically active by Ca^2+^/calmodulin dependent conversion of L-Arginine to L-citrulline [8] and to be tightly regulated by arginase-1 [9], its impact on HbNO accumulation in RBCs was not analyzed before. Our data show that, under inhibition of Arginase-1, the erythrocyte eNOS contributes to the formation of HbNO, measured by EPR in RBC *ex vivo*, in parallel to L-NAME sensitive intracellular accumulation of nitrite and nitrate. As negative control, the HbNO signal was insensitive to nor-NOHA and L-NAME in the RBCs from eNOS^(-/-)^ mice. Residual nitrite and nitrate formation can be explained by the presence of oxygenated hemoglobin that converted NO into oxidized metabolites [36]. Therefore the HbNO complex can reflect eNOS activity in RBCs under Arginase-1 inhibition *ex vivo* [9]]. We next examined the contribution of erythrocyte NO production on the levels of venous HbNO pre-formed *in vivo*. Even though RBCs express an active eNOS, the functionality of RBC-derived NO is still discussed (reviewed in [37]). Some studies suggested that NO, derived from the erythrocyte eNOS, may protect the vasculature from ischemia-reperfusion injury [9], regulate blood pressure [12], inhibit platelet activation and aggregation [8] and promote hypoxic vasodilation [38]. However, some recent studies disputed the involvement of erythrocytes in NO-dependent platelet inhibition [13] or hypoxic vasodilatation through activation of soluble guanylyl cyclase in platelets and smooth muscle cells, respectively (reviewed in [37]). A soluble guanylate cyclase was recently shown to operate in RBCs, accounting for increased cGMP production upon stimulation of erythrocytes with NO [39]. cGMP-induced Protein Kinase G (PKG) activity in RBCs was proposed to regulate erythrocyte membrane properties and RBC clearance. Accordingly, PKG–deficient mice are anemic [40, 41]. NO itself was also proposed as a modulator of RBCs deformability and membrane fluidity. In fact, inhibition of NO formation by NOS inhibitors *in vivo* drastically reduced RBC deformability, whereas low concentrations of NO donors affect membrane fluidity and increase RBC deformability (reviewed in [42]). However, these NO donors may protect erythrocytes only against calcium-induced loss of deformability or in sickle cell disease patients after deoxygenation-induced erythrocyte dehydration [43, 44]. Moreover, others have shown that NO only preserves erythrocyte deformability in increased oxidative stress conditions, with no effect on blood bulk viscosity in unstressed conditions. Indeed, erythrocytes from eNOS^(-/-)^ mice showed deformability fully comparable to wild-type under matched intracellular ROS level [45]. NO can additionally be oxidized into nitrate by oxyhemoglobin in RBCs or forms nitrosothiols (and S-nitrosohemoglobin) in redox S-nitrosation, and trans-nitrosation reactions during RBC oxygenation/deoxygenation cycles *in vivo* as was demonstrated previously [10]. However, the true involvement of erythrocyte NOS remained dubious. Our data show that the erythrocyte HbNO signal, formed *in vivo* and sequentially measured *ex vivo* decreased only minimally after treating RBCs with L-NAME under normoxia or hypoxic conditions up to 40 minutes. Even nor-NOHA pre-treatment of erythrocytes only minimally changed the HbNO stability under these conditions (Fig 6). These observations suggest that the effect of erythrocyte NOS on venous HbNO content is marginal despite its ability to produce NO in oxygenated conditions [7]. Alternatively, we would propose that most of HbNO is formed from vascular or endothelial NO reaching circulating RBCs and binding intracellular (or membrane associated) hemoglobin. Nevertheless the erythrocyte membrane may have limited permeability to NO produced extracellularly by NO-donors as suggested in our (Fig 1) and a previous study [46]. NO consumption by intact erythrocytes was shown to be slower than by free Hb suggesting that transmembrane and intracellular resistance of RBC act as rate-limiting factors for its diffusion into erythrocytes (reviewed in [42]). In support of our hypothesis, the administration of L-NAME *in vivo* (in drinking water of rats and mice), contrary to treatment of erythrocytes *ex vivo*, significantly decreased the HbNO EPR signal emphasizing the crucial role of vascular NOS activity. To more directly evaluate the specific contribution of the vascular endothelium to the HbNO content, we used a genetic mouse model deficient in caveolin-1, a well-established allosteric inhibitor of endothelial eNOS. Using this model, we previously demonstrated a strong potentiation of calcium-induced eNOS activation in dissected aortas from cav-1^(-/-)^ mice, despite unchanged eNOS protein abundance [17]. As we could not identify any measurable expression of cav-1 in erythrocytes from wild-type animals (S7A and S7B Fig), cav-1 genetic deletion would only be expected to affect eNOS activity in the vascular wall, not in erythrocytes. Nevertheless, the venous HbNO content was clearly increased (by~70%) in freshly drawn blood from cav-1^(-/-)^ mice compared with their wild-type littermate, corroborating the idea that erythrocyte HbNO content mostly reflects NO bioavailability from the vascular wall. This is in line with recent independent observations of increased HbNO in endotoxemic rodents after iNOS induction by lipopolysaccharide injection [47]. As mature erythrocytes are anucleated and cannot express iNOS upon acute induction [7], their HbNO content again mainly originates from external (e.g. mostly vascular) sources in this context. In support of this, *in vivo* administration of isosorbide dinitrate, a potent nitrovasodilator and NO donor ([48] and reviewed in [49]), produced clearly measurable increases in erythrocyte HbNO level in our hands (Fig 7). The dynamic variation of the signal highlights the sensitivity of erythrocyte HbNO to the fast reaction with NO produced in the vasculature. This may be extended upon administration of high doses of nitrite (>20mg orally) or nitrate-enriched diet (with subsequent reduction to nitrite by enterosalivary microbes), both of which also can generate NO and increase HbNO [48].

In conclusion we demonstrated that the erythrocyte eNOS has a constitutive activity but that the NO produced only minimally influences the whole HbNO formation in erythrocytes, suggesting that the HbNO complex mainly results from, and may reflect the bioavailability of NO formed in the vasculature. Our further characterization of HbNO stability formed in intact erythrocytes under biochemical conditions reflecting the vascular milieu will help to standardize the measurements of this paramagnetic compound as a surrogate of circulating nitrosylating species.

## Supporting information

S1 Fig A. EPR spectra and B. calibration curve were obtained after addition of different concentration of NO-donor system (0–0.5–1–2–4–8 μmol/L of NO_2_^-^ and dithionate Na_2_S_2_O_4_) to human RBCs under hypoxic condition. Samples were frozen in calibrated tubes for low-temperature EPR measurements as described in Material and Methods. The three arrows indicate the hyperfine components (hfs) typical of the 5 coordinate HbNO; A(I) indicates the amplitude of the first component.
(TIF)

S2 Fig A. EPR spectra of RBCs pre-incubated with a NO-donor at venous O_2_ level (45 minutes) and exposed to re-oxygenation (21% of O_2_) for 1 hour (0–60 min.). (Upper) a0-a60 are EPR spectra of HbNO formed in venous RBC at time 0 (a0) and over time at 21% O_2_ (a20-a60); (Lower) a0-xA to a60-xA are residual EPR spectra at time 0 (a0-xA) and over time at 21%O_2_ (a20-xA to a60-xA) after subtraction of the model spectrum of alpha-5-coordinate heme Fe(II)-HbNO (represented in A, below) to unveil additional nitrosylated Hb in R form; the spectrum of HbNO formed in oxygenated blood (R-form) is shown for comparison. B. Decay of HbNO EPR signal recorded in human erythrocytes incubated until 50 minutes in presence (open square) or absence (closed square) of a column of plasma at 21% O_2_ or without plasma at 1% O_2_ (open triangle). Data are shown as mean ± SD; ^*^ P < 0.05; n = 4 different preparations of RBCs.
(TIF)

S3 Fig Effect of pH influence on HbNO concentrations in human RBCs pre-incubated with NO-donor and reconstituted at 50% haematocrit with buffer at physiological pH (7.4–7.2) or at acidic pH (6.2–4.7) during incubation in hypoxic condition (1% of O_2_). Data are shown as mean values ± SEM; ^*^ P < 0.05; n = 4 RBCs different preparations.
(TIF)

S4 Fig Immunoprecipitation followed by western blot analysis revealed an eNOS-specific band at 130 kDa in A. eNOS^(+/+)^ mouse and B. in human RBCs and membranes. This band is equivalent to positive controls of mouse lung extracts and of human aortic endothelial cells. eNOS^(-/-)^ mice RBC and membrane do not show eNOS expression (A). C. Representative flow cytometric two parameter dot plot of isolated rat RBCs co-stained with primary anti-Aquaporin-1 and secondary Alexa Fluor-488-conjugated anti-IgG antibodies; and primary anti-eNOS and secondary Alexa Fluor-647-conjugated anti-IgG antibodies. Panel on the left shows co-staining only with conjugated secondary antibodies (negative control). Panel on the right shows the identification of R as eNOS–positive and Aquaporin-1 positive events in the upper right quadrant. The percentage of RBCs double positive is indicated in the upper right quadrant.
(TIF)

S5 Fig Effect of NOS and/or arginase inhibition *ex vivo* on HbNO content in rat erythrocytes. Decay of HbNO EPR signal formed in rat erythrocytes *in vivo* after *ex vivo* treatment with L-NAME or vehicle during incubation at 4% (A) or 21% of O_2_ (B) or with nor-NOHA at 21% of O_2_ (C) under L-Arginine supplementation as described in Material and Methods. Samples were frozen every 5 minutes until 40 minutes for low-temperature EPR measurements. Data are shown as mean values ± SEM; ^*^ P < 0.05; n = 6 RBCs different preparations.
(TIF)

S6 Fig HbNO sensitivity to *in vivo* administered L-NAME. Concentration of HbNO in venous rat RBCs after one and seven days of treatment with L-NAME or vehicle. Data are shown as mean values ± SD; ^*^ P < 0.05; n = 4 RBCs different preparations.
(TIF)

S7 Fig Absence of caveolin-1 expression in RBCs from wild type mice and comparative HbNO levels in erythrocytes from cav-1^(+/+)^ and cav-1^(-/-)^ mice. Representative immunoblotted signal for caveolin-1, as detected in mouse lung extracts (with rich endothelial cells content, positive control), but undetectable in whole erythrocyte lysates, cytosolic fractions (A) or ghost membranes (B) from wild-type mice, despite loading with excess erythrocyte total proteins (as visible from Ponceau red staining of the gels; RBC: 80μg loaded; lung: 10μg loaded; A and B). HbNO concentrations (C) and typical EPR spectra (D) of venous erythrocyte HbNO freshly drawn from cav-1^(+/+)^ and cav-1^(-/-)^ mice. Data are shown as mean values ± SD; ^*^ P < 0.05; n = 4 different preparations from 4 mice each.
(TIF)
