# Supplementary figures and images for: Nitrosyl-hemoglobin formation in rodent and human venous erythrocytes reflects NO formation from the vasculature in vivo
Source: PLoS One. 2018 Jul 11;13(7):e0200352. doi: 10.1371/journal.pone.0200352 (PMC6040712; doi:10.1371/journal.pone.0200352)

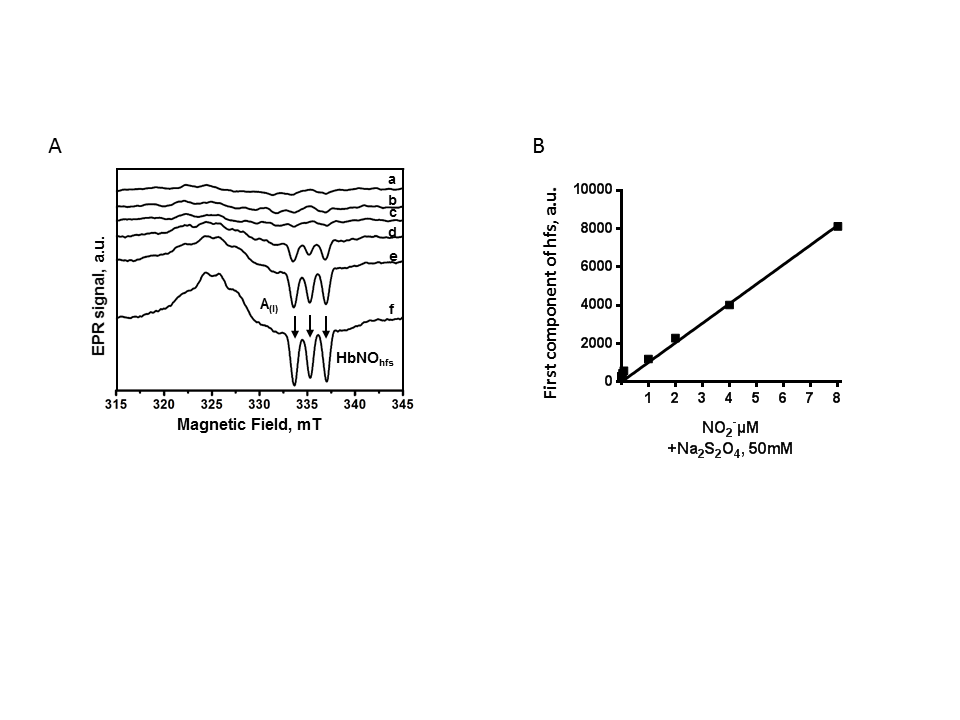

Supplement: S1 Fig — A. EPR spectra and B. calibration curve were obtained after addition of different concentration of NO-donor system (0–0.5–1–2–4–8 μmol/L of NO2- and dithionate Na2S2O4) to human RBCs under hypoxic condition. Samples were frozen in calibrated tubes for low-temperature EPR measurements as described in Material and Methods. The three arrows indicate the hyperfine components (hfs) typical of the 5 coordinate HbNO; A(I) indicates the amplitude of the first component. (TIF) [file pone.0200352.s001.tif]

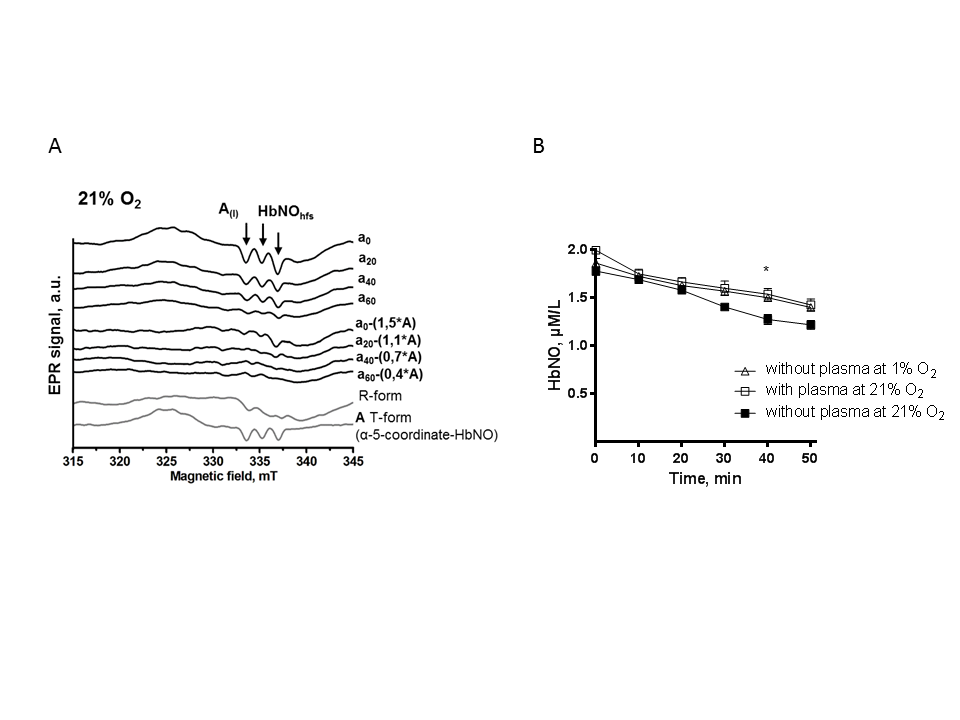

Supplement: S2 Fig — A. EPR spectra of RBCs pre-incubated with a NO-donor at venous O2 level (45 minutes) and exposed to re-oxygenation (21% of O2) for 1 hour (0–60 min.). (Upper) a0-a60 are EPR spectra of HbNO formed in venous RBC at time 0 (a0) and over time at 21% O2 (a20-a60); (Lower) a0-xA to a60-xA are residual EPR spectra at time 0 (a0-xA) and over time at 21%O2 (a20-xA to a60-xA) after subtraction of the model spectrum of alpha-5-coordinate heme Fe(II)-HbNO (represented in A, below) to unveil additional nitrosylated Hb in R form; the spectrum of HbNO formed in oxygenated blood (R-form) is shown for comparison. B. Decay of HbNO EPR signal recorded in human erythrocytes incubated until 50 minutes in presence (open square) or absence (closed square) of a column of plasma at 21% O2 or without plasma at 1% O2 (open triangle). Data are shown as mean ± SD; * P < 0.05; n = 4 different preparations of RBCs. (TIF) [file pone.0200352.s002.tif]

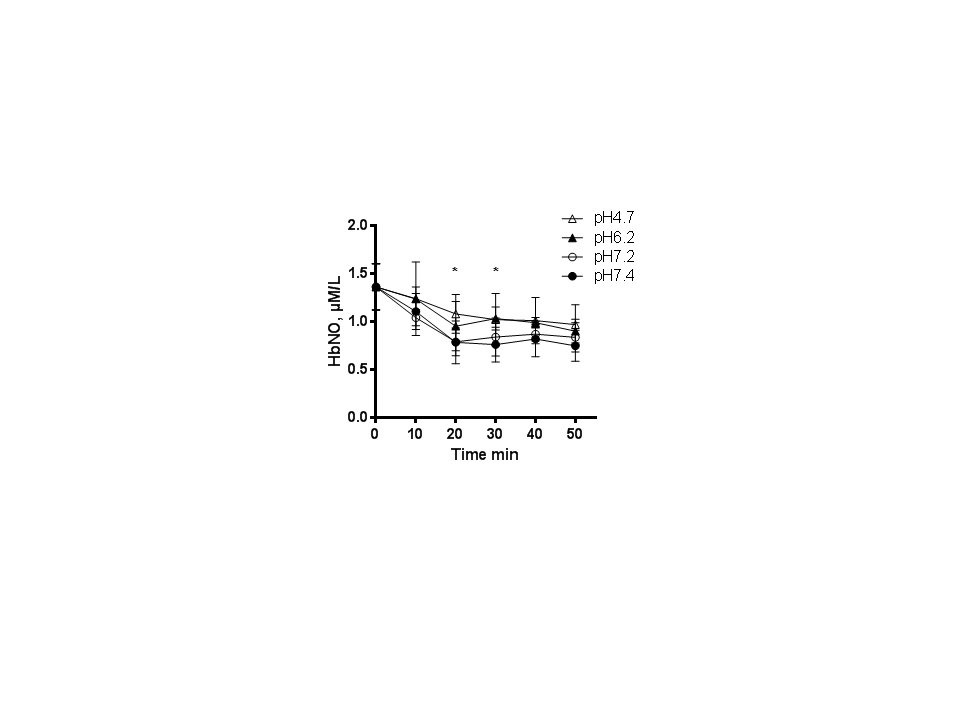

Supplement: S3 Fig — Data are shown as mean values ± SEM; * P < 0.05; n = 4 RBCs different preparations. (TIF) [file pone.0200352.s003.tif]

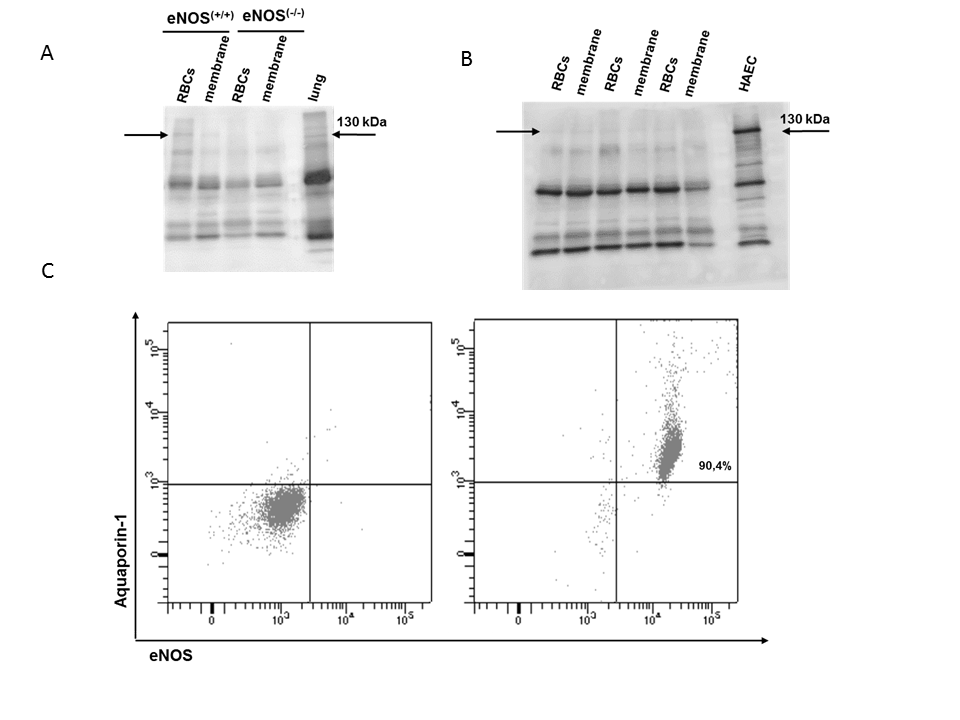

Supplement: S4 Fig — This band is equivalent to positive controls of mouse lung extracts and of human aortic endothelial cells. eNOS(-/-) mice RBC and membrane do not show eNOS expression (A). C. Representative flow cytometric two parameter dot plot of isolated rat RBCs co-stained with primary anti-Aquaporin-1 and secondary Alexa Fluor-488-conjugated anti-IgG antibodies; and primary anti-eNOS and secondary Alexa Fluor-647-conjugated anti-IgG antibodies. Panel on the left shows co-staining only with conjugated secondary antibodies (negative control). Panel on the right shows the identification of R as eNOS–positive and Aquaporin-1 positive events in the upper right quadrant. The percentage of RBCs double positive is indicated in the upper right quadrant. (TIF) [file pone.0200352.s004.tif]

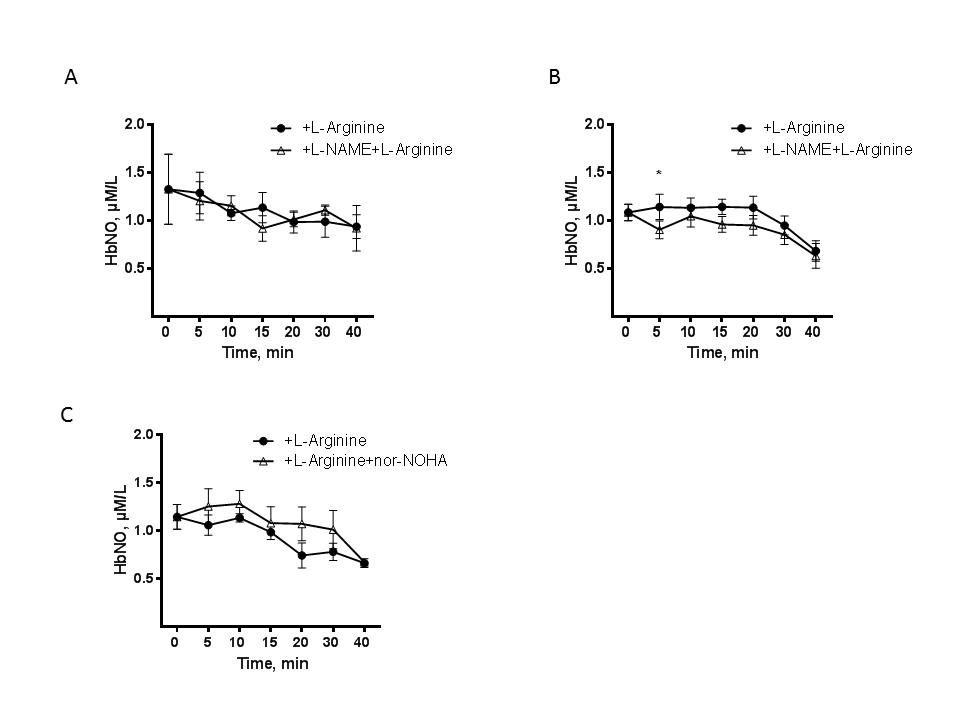

Supplement: S5 Fig — Decay of HbNO EPR signal formed in rat erythrocytes in vivo after ex vivo treatment with L-NAME or vehicle during incubation at 4% (A) or 21% of O2 (B) or with nor-NOHA at 21% of O2 (C) under L-Arginine supplementation as described in Material and Methods. Samples were frozen every 5 minutes until 40 minutes for low-temperature EPR measurements. Data are shown as mean values ± SEM; * P < 0.05; n = 6 RBCs different preparations. (TIF) [file pone.0200352.s005.tif]

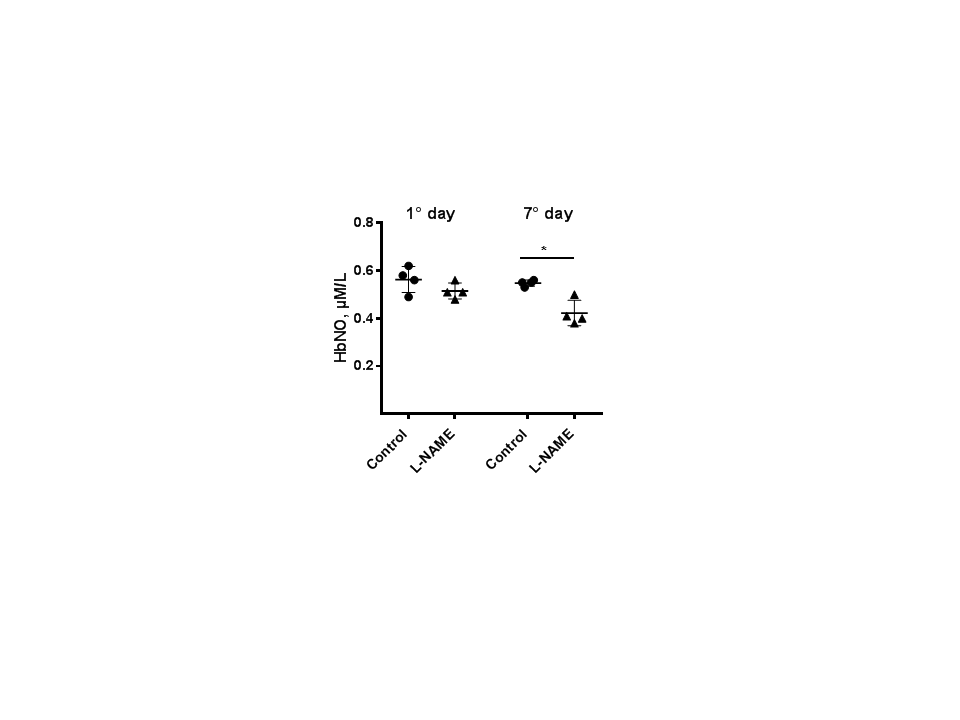

Supplement: S6 Fig — Concentration of HbNO in venous rat RBCs after one and seven days of treatment with L-NAME or vehicle. Data are shown as mean values ± SD; * P < 0.05; n = 4 RBCs different preparations. (TIF) [file pone.0200352.s006.tif]

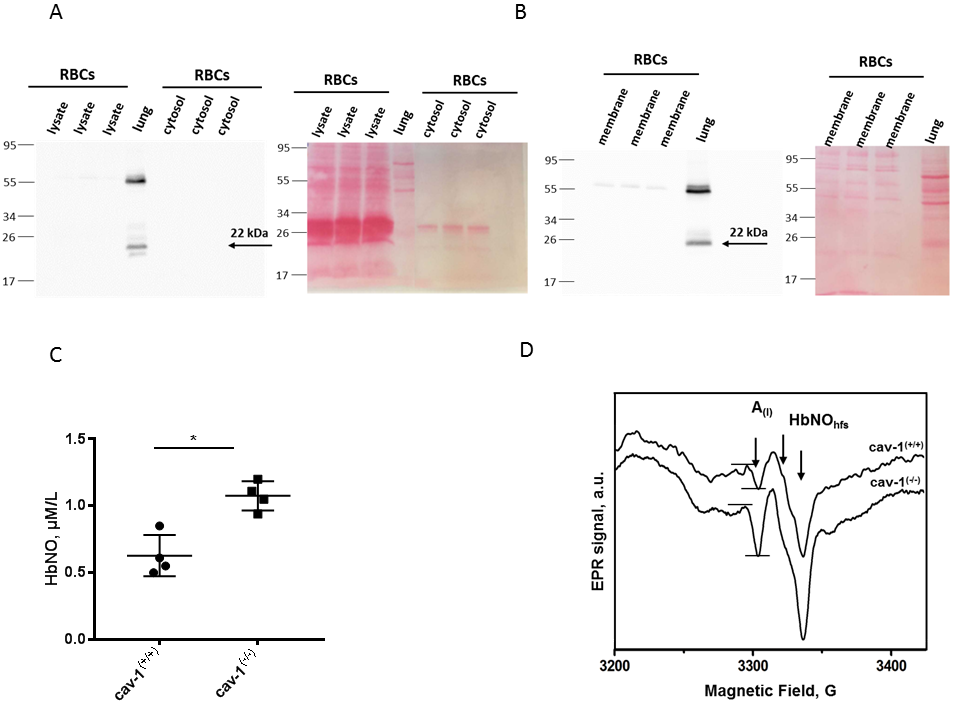

Supplement: S7 Fig — Representative immunoblotted signal for caveolin-1, as detected in mouse lung extracts (with rich endothelial cells content, positive control), but undetectable in whole erythrocyte lysates, cytosolic fractions (A) or ghost membranes (B) from wild-type mice, despite loading with excess erythrocyte total proteins (as visible from Ponceau red staining of the gels; RBC: 80μg loaded; lung: 10μg loaded; A and B). HbNO concentrations (C) and typical EPR spectra (D) of venous erythrocyte HbNO freshly drawn from cav-1(+/+) and cav-1(-/-) mice. Data are shown as mean values ± SD; * P < 0.05; n = 4 different preparations from 4 mice each. (TIF) [file pone.0200352.s007.tif]
